# Association of the single-point insulin sensitivity estimator (SPISE) index and incident cardiovascular disease in early-stage CKM syndrome: insights from the UK biobank

**DOI:** 10.1186/s13098-026-02222-z

**Published:** 2026-06-25

**Authors:** Jie Zou, Zhoujian Han, Yuexin Pan, Zeru Chen, Hongyu Zhong, Rongshuai Pan, Jingquan Zhou, Jie Xiao, Xiaoqian Wu, Mingyan Li

**Affiliations:** 1 Department of Cardiology, Guangdong Key Laboratory of Vascular Diseases, The Second Affiliated Hospital, Guangzhou Institute of Cardiovascular Disease, Guangzhou Medical University, Guangzhou, Guangdong China; 2 The Second School of Clinical Medicine, Guangzhou Medical University, Guangdong, Guangzhou China; 3 Cardiometabolic Medicine Center, Fuwai Hospital, National Center for Cardiovascular Diseases, Chinese Academy of Medical Sciences and Peking Union Medical College, Beijing, China; 4 Guangdong Provincial Key Laboratory of Molecular Target & Clinical Pharmacology, the NMPA and State Key Laboratory of Respiratory Disease, the School of Pharmaceutical Sciences, Guangzhou Medical University, Guangzhou, Guangdong China; 5 The First School of Clinical Medicine, Guangzhou Medical University, Guangzhou, Guangdong China

**Keywords:** Cardiovascular disease, Cardiovascular-kidney-metabolic syndrome, Single-point insulin sensitivity estimator, Insulin resistance

## Abstract

**Background:**

Insulin resistance is a core metabolic abnormality driving the progression of cardiovascular-kidney-metabolic (CKM) syndrome. However, the gold-standard hyperinsulinemia-euglycemic clamp remains challenging for routine clinical use. The Single-Point Insulin Sensitivity Estimator (SPISE) index, calculated from HDL-C, triglycerides, and BMI without requiring insulin measurement, offers a simple and noninvasive alternative. However, its prognostic value for incident cardiovascular disease (CVD) in early-stage CKM syndrome (stages 0–3) has not been established.

**Methods:**

This prospective cohort study included 391,793 participants from the UK Biobank with CKM stage 0–3. Participants were followed for a median of 7.6 years. Cox proportional hazards regression, and restricted cubic spline regression analyses were employed to examine the association between SPISE and incident CVD and coronary heart disease (CHD), adjusting for demographic, lifestyle, and clinical covariates. Subgroup analyses and sensitivity analyses were also conducted.

**Results:**

During follow-up, 66,577 participants (17%) with stage 0–3 CKM syndrome experienced incident CVD, including 31,597 cases (8%) of CHD. SPISE demonstrated a significant L-shaped, inverse association with CVD risk, with an inflection point at 5.81 (P for nonlinearity < 0.001).Compared with the lowest tertile, the second and highest tertiles of SPISE were associated with 21% (HR 0.79, 95% CI 0.77–0.80) and 31% (HR 0.69, 95% CI 0.67–0.70) lower risk of incident CVD, respectively. The protective association was stronger for CHD, with corresponding HRs of 0.73 (95% CI 0.71–0.75) and 0.54 (95% CI 0.52–0.55). Subgroup analyses revealed more pronounced benefits among younger participants (< 45 years; HR 0.71, 95% CI 0.68–0.74 for CVD) and never/previous smokers (P for interaction < 0.005).

**Conclusion:**

In this large population-based study, higher SPISE index was independently and nonlinearly associated with lower risk of incident CVD and CHD in patients with early-stage CKM syndrome. The SPISE index may serve as a practical, cost-effective tool for cardiovascular risk stratification and early prevention in this high-risk population.

**Graphical abstract:**

**Figure:**
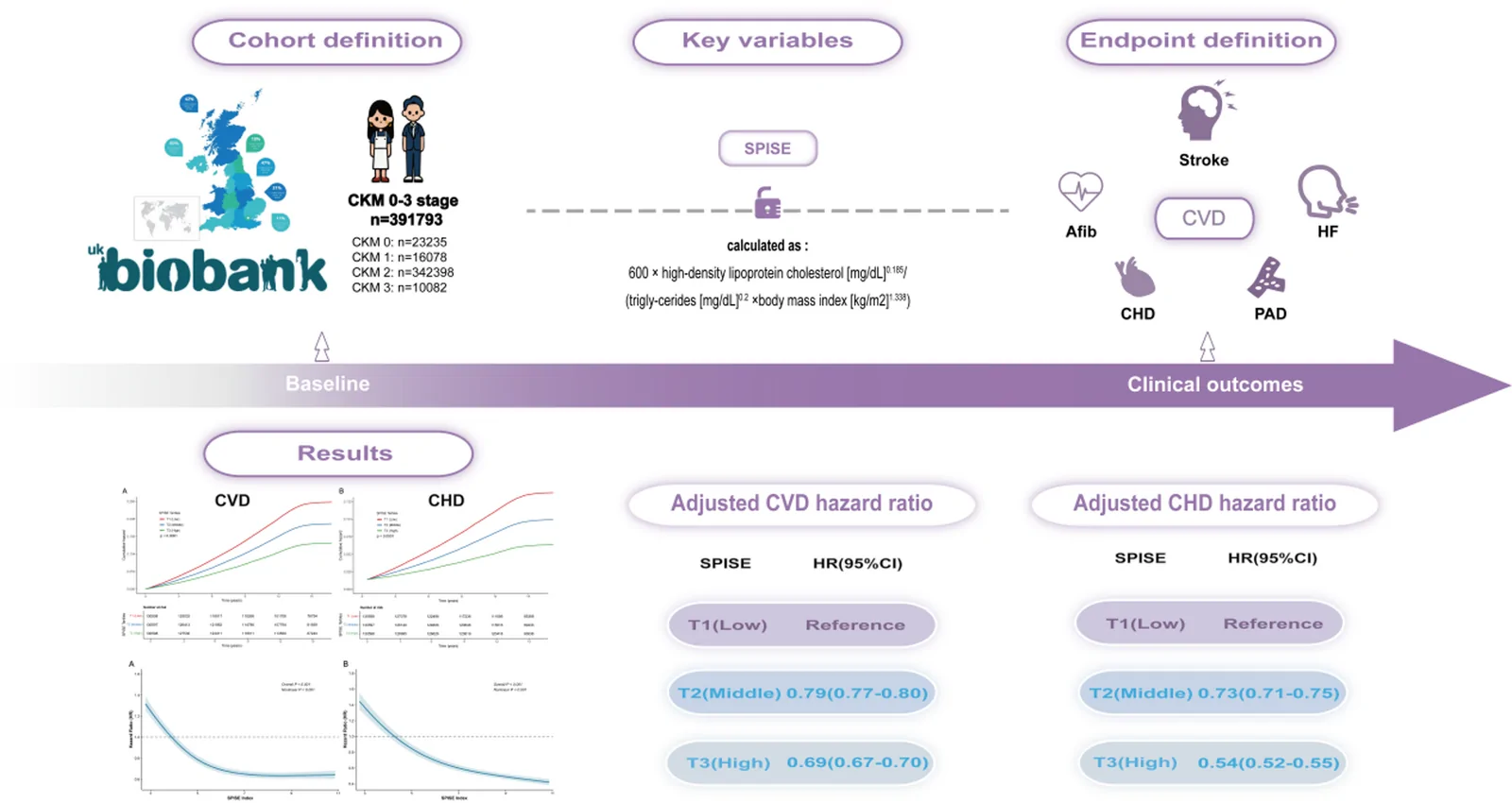

**Supplementary Information:**

The online version contains supplementary material available at https://doi.org/10.1186/s13098-026-02222-z.

## Introduction

Cardiovascular–Kidney–Metabolic (CKM) syndrome reflects the close pathophysiological interconnections among cardiovascular disease (CVD), chronic kidney disease (CKD), and metabolic disorders, such as obesity and type 2 diabetes. The framework proposed by the American Heart Association (AHA) classifies CKM syndrome into five stages, ranging from stage 0 (no CKM risk factors) to stage 4 (clinical CVD in CKM syndrome) [1]. Epidemiological data indicate that nearly 90% of U.S. adults meet the criteria for CKM syndrome at stage 1 or higher, and approximately 15% have progressed to advanced stages [2]. Moreover, accumulating evidence demonstrates a stepwise increase in cardiovascular and all-cause mortality with advancing CKM stage, highlighting the prognostic significance of CKM even at its early stages [3, 4]. Therefore, strengthening primary prevention of cardiovascular disease—particularly among the general population, individuals with subclinical cardiovascular disease, and those with high-risk metabolic profiles—is critical to delaying CKM progression and reducing future cardiovascular events [5].

Insulin resistance (IR) is defined as an impaired ability of insulin to regulate glucose metabolism in target tissues and is closely associated with multiple chronic conditions, including metabolic syndrome, cardiovascular disease, and type 2 diabetes [6–8]. Existing evidence suggests that, during the progression of CKM syndrome, IR serves as a core metabolic abnormality that promotes organ damage through several interconnected mechanisms, including chronic inflammation, endothelial injury, glomerular hyperfiltration, and alterations in cardiac energy metabolism [9–11]. Accordingly, accurate assessment of IR is essential for early risk identification and primary prevention of CKM syndrome. Although the hyperinsulinemic-euglycemic clamp remains the gold standard for evaluating IR, its invasiveness, high cost, and technical complexity limit its widespread application [12]. There is an increasing demand for reliable, non-invasive, and easily accessible tools to assess IR.

The Single-Point Insulin Sensitivity Estimator (SPISE) index offers a novel approach for assessing IR, as it is derived from high-density lipoprotein cholesterol (HDL-C), triglycerides (TG), and body mass index (BMI) without requiring insulin measurements [13]. Recent systematic reviews have demonstrated that SPISE outperforms several traditional IR indices, such as HOMA-IR, the TG-to-HDL cholesterol ratio, and the triglyceride–glucose index (TyG), in screening for metabolic syndrome. In addition, multiple studies have reported significant associations between SPISE and adverse cardiovascular outcomes in individuals with type 2 diabetes, as well as the risk of heart failure in middle-aged and older adults, underscoring its potential as an integrated marker of metabolic and cardiovascular health [14–17]. However, systematic evidence regarding the association between SPISE and incident cardiovascular disease in the early stages of CKM syndrome remains limited. Clarifying this relationship may provide important insights into how insulin sensitivity mediates CKM-related cardiovascular risk and support the identification of novel predictive markers. Accordingly, the present study aimed to investigate the association between the SPISE index and incident cardiovascular disease in the early stages of CKM syndrome using data from the UK Biobank (UKB).

## Methods

### Data Sources and study population

UKB is a large-scale prospective cohort of over 500,000 UK adults aged 37–73 at recruitment (2006–2010), integrating questionnaires, physical measurements, biosamples, and multimodal follow-up including imaging examinations and detailed follow-up assessments [18, 19]. The sufficient sample size and coverage of critical cardiovascular exposures necessitated the evaluation of the association between the SPISE index and cardiovascular events in early-stage CKM syndrome patients. Ethical approval for the UKB project was granted by the Northwest Multi-centre Research Ethics Committee (MREC reference: 21/NW/0157). All participants provided informed consent prior to enrollment.

**Fig. 1:**
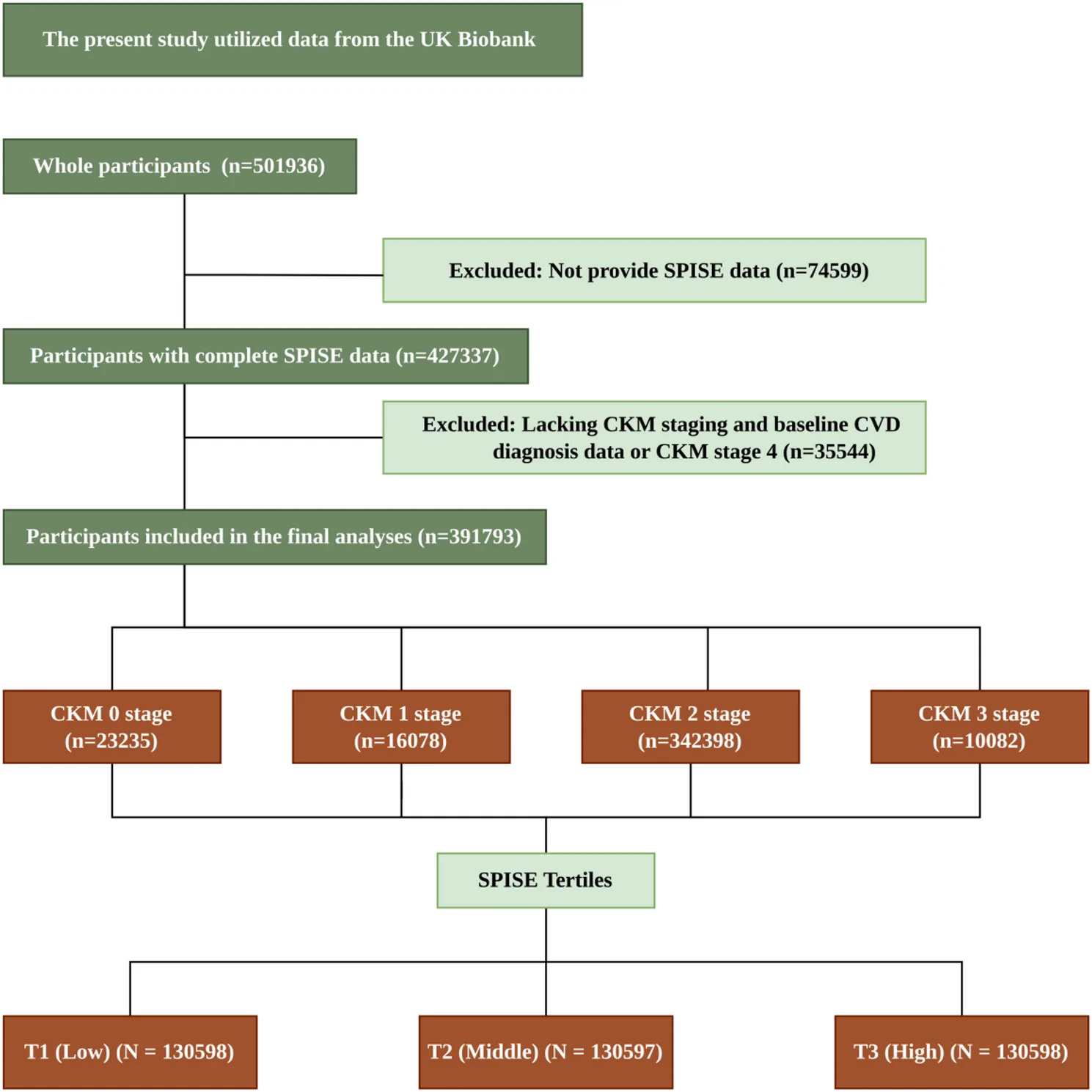
A schematic diagram of the participant inclusion and exclusion process

The initial cohort comprised 501,936 participants. Those lacking data required for SPISE calculation (*n* = 74,599) were excluded from the analysis cohort. Subsequently, participants missing data for CKM staging, those with baseline CVD diagnosis, and patients diagnosed with CKM stage 4 (*n* = 35,544) were excluded. Following these exclusion criteria, the final analysis cohort comprised 391,793 individuals with representative characteristics of CKM stages 0–3 (Fig. 1).

The proportion of missing data for key covariates is relatively low. Missing rates are as follows: glycated hemoglobin (HbA1c) (5.19%), alcohol consumption (4.48%), education level (1.03%), smoking status (0.47%), ethnicity (0.36%), insomnia (0.21%), C-reactive protein (CRP) (0.21%), Townsend score (0.12%), EGFR status (0.07%), and cholesterol (0.01%). To minimize potential bias and improve the accuracy of model estimates, missing values were imputed using multiple imputation by chained equations (MICE). Predictive mean matching (PMM) was used for continuous variables, proportional odds logistic regression (polr) for ordinal categorical variables, logistic regression (logreg) for binary variables, and polytomous regression (polyreg) for unordered categorical variables with more than two categories.

### Definition of CKM syndrome and SPISE index

The concept of CKM syndrome was introduced and defined by the AHA in 2023, resulting from the interconnection between obesity, diabetes, CKD, and CVD, including heart failure (HF), atrial fibrillation (AF), coronary heart disease (CHD), stroke, and peripheral arterial disease (PAD). It is categorized into five progressive stages [1]. In this study, the CKM staging of patients was adjusted according to the available variables in the UKB database, with specific criteria referenced in the literature [18]. See Table S1 for details.

In this study, the baseline SPISE index was used as the predictor and was calculated as 600 × high-density lipoprotein cholesterol [mg/dL]^0.185^/(trigly-cerides [mg/dL]^0.2^ ×body mass index [kg/m^2^]^1.338^) [13, 17].

### Data collection

#### Assessment of CVD

Consistent with the AHA criteria for CKM stage 4, the diagnosis of CVD in this study was determined based on hospitalization records and death registry data, including HF, AF, CHD, stroke, or PAD. For specific UKB codes, refer to the literature [18]. See the Table S2 for specific criteria.

#### Covariates

This study included covariates covering sociodemographic characteristics, lifestyle factors, and previous determinants of cardiovascular disease risk as well as potential confounders in exposure-outcome relationships. Self-reported data via touchscreen questionnaires included baseline age, gender, ethnicity, education level, alcohol consumption status, smoking status, insomnia frequency, dietary quality index, the Thomson Poverty Index, and physical activity level. Trained nurses measured anthropometric waist circumference, with BMI calculated as weight (kg)/height (m)². Biochemical analysis of baseline blood samples quantified HDL-C, TG, total cholesterol (TC), fasting blood glucose (FBG), HbA1c, CRP, urinary creatinine, urinary albumin, serum creatinine, and natriuretic peptide C. Nurse interviews assessed medical history of hypertension, diabetes, and dyslipidemia, along with medication use (including lipid-lowering agents, antihypertensive drugs, and antidiabetic medications). Detailed definitions of these variables are provided in Supplementary Table S3-5.

#### Statistical analysis

Participants were divided into three tertiles based on their SPISE scores, and baseline characteristics were summarized according to these tertiles. Continuous variables are expressed as mean [standard deviation (SD)], and categorical variables are presented as frequency (percentage). Cumulative risk curves estimated cumulative cardiovascular disease incidence, with log-rank tests assessing intergroup differences. Cox proportional hazards models quantified the association between SPISE scores and cardiovascular disease risk, expressed as hazard ratios (HR) with 95% confidence intervals (CI). Survival curves based on Cox models illustrated cumulative risk differences across SPISE tertiles. Three sequential adjustment models were implemented: Model 1 considered only SPISE tertiles, sex, and age; Model 2 adjusted for demographic and lifestyle covariates (age, sex, race, education level, Townsend poverty index, alcohol consumption, smoking status, dietary score); Model 3 further adjusted for medications (lipid-lowering, antihypertensive, antidiabetic drugs). Collinearity tests (Table S6) revealed variance inflation factors < 5 for each covariate, indicating no significant multicollinearity issues. Model 3 was selected as optimal for subsequent analyses based on Akaike and Bayesian information criterion (AIC/BIC) scores (Table S7).

Additionally, four restricted cubic splines (RCS) were fitted to assess the nonlinear association between the SPISE index and CVD incidence rate. Subgroup analyses were subsequently conducted to investigate whether the association between cardiovascular disease incidence and the SPISE index differed across varying levels of covariates, including gender, age, ethnicity, education level, smoking and drinking status, hypertension, and diabetes.

To validate the robustness of our findings, we conducted sensitivity analyses. First, we validated the results by adjusting for a broader range of potential confounders. Second, to exclude potential reverse causality, we excluded participants who experienced CVD or CHD events within the first 3 years of follow-up. Third, we assessed potential selection bias due to data missingness by excluding samples with incomplete covariate data. Fourth, in subgroup analyses, the exposure variable was re-stratified into quartiles. Using the lowest quartile (Q1) as the reference, we assessed the risk ratios for Q2–Q4 and performed trend tests (P for trend).

All analyses were performed using R version 4.4.3. In all statistical analyses, a two-sided p-value < 0.05 was considered statistically significant.

## Results

### Baseline characteristics of study participants

Table 1 and Table S8 presents baseline characteristics of 391,793 individuals with CKM syndrome stages 0–3, including 174,487 men (45%) and 217,306 women (55%), with a mean age (SD) of 56.1 years (8.1 years). During follow-up, 66,577 participants experienced cardiovascular events, including 31,597 cases of CHD. Stratified by SPISE tertiles, High SPISE was associated with younger age, higher proportion of females, and lower educational attainment (*P* < 0.001). Regarding lifestyle factors, high SPISE levels were associated with higher proportions of never smokers, current drinkers, dietary scores of 3, occasional insomnia, and adequate physical activity (*P* < 0.001). Notably, while high SPISE—indicative of a more favorable metabolic profile—was associated with a greater proportion of current drinkers, the prevalence of current alcohol consumption remained consistently high across all SPISE tertiles. Biochemically, individuals with High SPISE exhibited higher HDL-C levels and lower TG, TC, FBG, HbA1c, CRP, and eGFR levels (all *P* < 0.001). Clinically, High SPISE participants exhibited favorable metabolic characteristics, including lower BMI and Waist circumference (WC) levels, lower prevalence of diabetes, hypertension, and hyperlipidemia, and were associated with later and fewer CVD or CHD events (*P* < 0.001). Notably, the incidence of CVD events and CHD risk progressively decreased across tertiles, reaching the lowest levels in the highest tertile at 12% and 5%, respectively (all *P* < 0.001). This advanced subgroup exhibited low levels of IR, further underscoring the interplay among metabolic, renal, and cardiovascular factors in CKM syndrome (Table S8).

**Table 1:** Baseline characteristics of the study population by SPISE tertiles

Characteristic	Overall (*N* = 391793)^1^	T1 (Low) (*N* = 130598)^1^	T2 (Middle) (*N* = 130597)^1^	T3 (High) (*N* = 130598)^1^	*P* value^2^
Age, years	56.1 (8.1)	56.4 (7.9)	56.8 (8.0)	55.2 (8.2)	< 0.001
Townsend deprivation index	-1.4 (3.1)	-1.1 (3.2)	-1.5 (3.0)	-1.5 (3.0)	< 0.001
Body mass index, kg/m²	27.3 (4.7)	32.0 (4.3)	26.8 (2.0)	23.1 (2.0)	< 0.001
Waist circumference, cm	89.8 (13.3)	101.9 (10.7)	89.4 (8.0)	78.0 (8.1)	< 0.001
Systolic blood pressure, mmHg	137.8 (18.6)	142.1 (17.5)	138.8 (18.2)	132.6 (18.8)	< 0.001
Diastolic blood pressure, mmHg	82.5 (10.1)	85.9 (9.6)	82.7 (9.6)	78.8 (9.7)	< 0.001
HDL cholesterol, mg/dL	56.6 (14.7)	47.2 (10.3)	55.5 (11.8)	66.9 (14.5)	< 0.001
Triglycerides, mg/dL	153.7 (90.4)	221.1 (108.1)	144.9 (58.5)	95.0 (36.5)	< 0.001
Total cholesterol, mg/dL	223.1 (43.0)	224.1 (45.8)	225.3 (42.9)	219.8 (40.1)	< 0.001
Fasting blood glucose, mg/dL	91.7 (21.0)	95.6 (27.5)	90.7 (17.3)	88.8 (15.7)	< 0.001
HbA1c, %	5.4 (0.6)	5.6 (0.7)	5.4 (0.5)	5.3 (0.4)	< 0.001
Serum creatinine, mg/dL	0.8 (0.2)	0.8 (0.2)	0.8 (0.2)	0.8 (0.2)	< 0.001
C-reactive protein, mg/L	2.6 (4.3)	3.7 (4.7)	2.4 (4.0)	1.6 (3.7)	< 0.001
eGFR, mL/min/1.73 m²	95.6 (14.1)	91.5 (14.7)	95.3 (13.6)	100.0 (12.7)	< 0.001
SPISE index	6.0 (1.8)	4.2 (0.6)	5.8 (0.4)	8.1 (1.2)	< 0.001
Follow-up years (CVD)	7.6 (4.0)	7.5 (4.0)	7.7 (4.0)	7.9 (4.0)	< 0.001
Follow-up years (CHD)	7.7 (4.0)	7.6 (4.0)	7.7 (4.0)	7.8 (4.0)	< 0.001
**Sex**					< 0.001
Female	217,306 (55%)	58,491 (45%)	66,144 (51%)	92,671 (71%)	
Male	174,487 (45%)	72,107 (55%)	64,453 (49%)	37,927 (29%)	
**Ethnicity**					< 0.001
White	370,646 (95%)	123,246 (94%)	123,046 (94%)	124,354 (95%)	
Non-White	21,147 (5%)	7,352 (6%)	7,551 (6%)	6,244 (5%)	
**Education level**					< 0.001
College/uni+	45,955 (12%)	12,415 (10%)	15,263 (12%)	18,277 (14%)	
Below college	345,838 (88%)	118,183 (90%)	115,334 (88%)	112,321 (86%)	
**Smoking status**					< 0.001
Never	218,839 (56%)	67,390 (52%)	72,766 (56%)	78,683 (60%)	
Previous	132,097 (34%)	48,702 (37%)	44,314 (34%)	39,081 (30%)	
Current	40,857 (10%)	14,506 (11%)	13,517 (10%)	12,834 (10%)	
**Alcohol consumption**					< 0.001
Never	16,857 (4%)	6,669 (5%)	5,400 (4%)	4,788 (4%)	
Previous	13,098 (3%)	5,400 (4%)	3,966 (3%)	3,732 (3%)	
Current	361,838 (92%)	118,529 (91%)	121,231 (93%)	122,078 (93%)	
**Insomnia symptoms**					< 0.001
Never/Rarely	96,258 (25%)	31,126 (24%)	32,572 (25%)	32,560 (25%)	
Sometimes	188,040 (48%)	60,982 (47%)	63,381 (49%)	63,677 (49%)	
Usually	107,495 (27%)	38,490 (29%)	34,644 (27%)	34,361 (26%)	
**Physical activity**					< 0.001
Inadequate	100,474 (26%)	41,809 (32%)	31,868 (24%)	26,797 (21%)	
Adequate	291,319 (74%)	88,789 (68%)	98,729 (76%)	103,801 (79%)	
**Dietary score**					< 0.001
0	389 (0%)	173 (0%)	118 (0%)	98 (0%)	
1	341 (0%)	163 (0%)	96 (0%)	82 (0%)	
2	5,816 (1%)	2,655 (2%)	1,814 (1%)	1,347 (1%)	
3	385,247 (98%)	127,607 (98%)	128,569 (98%)	129,071 (99%)	
Hypertension	300,010 (77%)	116,644 (89%)	102,786 (79%)	80,580 (62%)	< 0.001
Diabetes	37,794 (10%)	24,770 (19%)	8,775 (7%)	4,249 (3%)	< 0.001
Dyslipidemia	279,688 (71%)	120,783 (92%)	98,021 (75%)	60,884 (47%)	< 0.001
Metabolic syndrome	118,056 (30%)	90,385 (69%)	24,930 (19%)	2,741 (2%)	< 0.001
Antihypertensive medication	38,279 (10%)	19,083 (15%)	12,344 (9%)	6,852 (5%)	< 0.001
Statin use	6,892 (2%)	2,928 (2%)	2,518 (2%)	1,446 (1%)	< 0.001
Antidiabetic medication	1,915 (0%)	1,048 (1%)	507 (0%)	360 (0%)	< 0.001
**CKM stage**					< 0.001
Stage 0	23,235 (6%)	0 (0%)	341 (0%)	22,894 (18%)	
Stage 1	16,078 (4%)	1,218 (1%)	7,434 (6%)	7,426 (6%)	
Stage 2	342,398 (87%)	122,443 (94%)	120,368 (92%)	99,587 (76%)	
Stage 3	10,082 (3%)	6,937 (5%)	2,454 (2%)	691 (1%)	
Incident cardiovascular disease	67,397 (17%)	28,846 (22%)	22,362 (17%)	16,189 (12%)	< 0.001
Incident coronary heart disease	32,174 (8%)	15,184 (12%)	10,678 (8%)	6,312 (5%)	< 0.001

^1^Continuous variables are presented as mean ± standard deviation. Categorical variables are presented as n (%). For binary variables, values represent the number (percentage) of ‘Yes’ cases

^2^One-way analysis of means (not assuming equal variances) for continuous variables; Pearson’s Chi-squared test for categorical variables

### Analyses of the correlation between the SPISE index (continous and tertiles) and CVD incidence

**Fig. 2:**
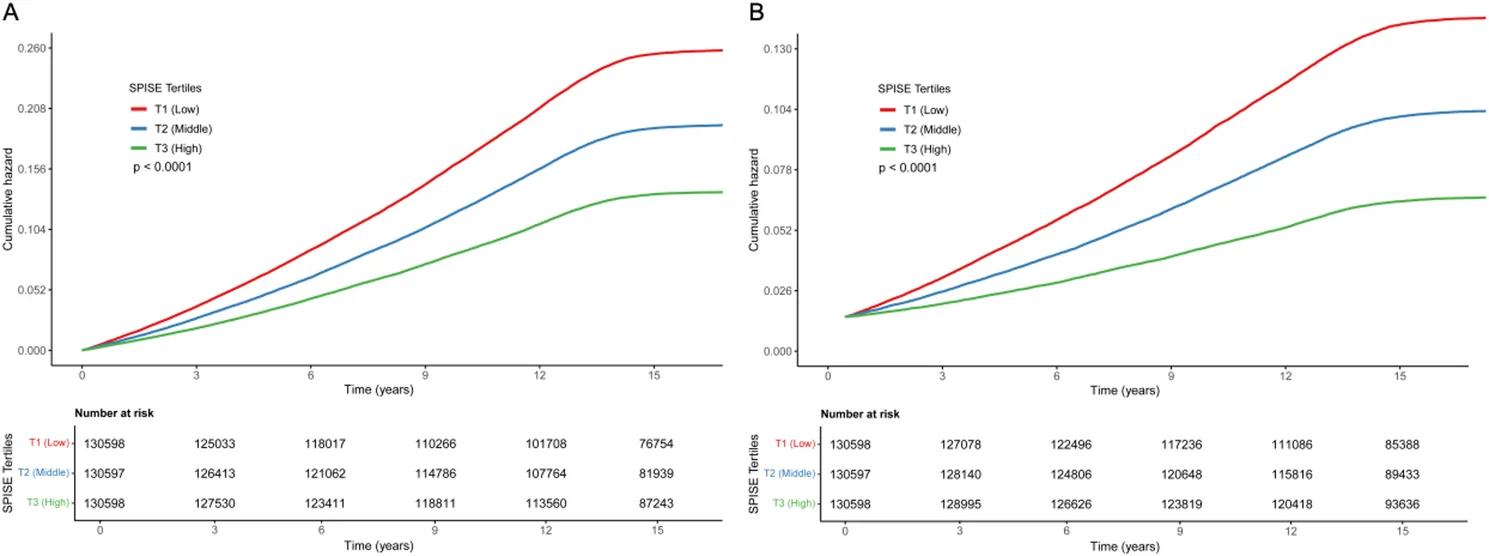
Cumulative risk curves for CVD (**A**) and CHD (**B**) risk across different SPISE tertiles in individuals with CKM stages 0–3. The global P value was calculated from the chi-square distribution with 2 degrees of freedom. SPISE, Single-Point Insulin Sensitivity Estimator; CKM, Cardiovascular-Kidney-Metabolic

Cumulative risk curves stratified by SPISE tertiles supported a dose-dependent protective relationship. The log-rank test revealed statistically significant differences in CVD incidence and CHD risk across tertiles (*P* < 0.05), with progressively lower CVD and CHD risks at higher SPISE levels (Fig. 2). After full adjustment for covariates, each 1-SD increase in SPISE index was associated with a 17% reduction in overall CVD risk (HR = 0.83, 95% CI 0.82–0.84) and a 27% reduction in CHD risk (HR = 0.74, 95% CI 0.73–0.75). From the perspective of SPISE tertiles, overall cardiovascular disease risk progressively decreased with increasing SPISE tertiles. Compared to the first tertile, the adjusted HR was 0.79 (95% CI 0.77–0.80) for the second tertile and 0.69 (95% CI 0.67–0.70) for the third tertile. A similar graded inverse association was observed for coronary heart disease, with HRs of 0.73 (95% CI 0.71–0.75) in the second tertile and 0.54 (95% CI 0.52–0.55) in the third tertile, indicating a statistically significant risk reduction (Table 2).

**Table 2:** Cox proportional hazards models for the association between SPISE index and CVD and CHD incidence

Exposures	Model 1 (HR, 95% CI)	*P*-value	Model 2 (HR, 95% CI)	*P*-value	Model 3 (HR, 95% CI)	*P*-value
**CVD**						
SPISE (per 1 SD)	0.80 (0.79–0.81)	< 0.001	0.82 (0.81–0.83)	< 0.001	0.83 (0.82–0.84)	< 0.001
SPISE (per 1 unit)	0.88 (0.88–0.89)		0.89 (0.89–0.90)		0.90 (0.90–0.91)	
T1 (Low)	1.00 (Reference)		1.00 (Reference)		1.00 (Reference)	
T2 (Middle)	0.75 (0.74–0.76)	< 0.001	0.77 (0.76–0.79)	< 0.001	0.79 (0.77–0.80)	< 0.001
T3 (High)	0.64 (0.62–0.65)	< 0.001	0.66 (0.65–0.68)	< 0.001	0.69 (0.67–0.70)	< 0.001
P for trend		< 0.001		< 0.001		< 0.001
**CHD**						
SPISE (per 1 SD)	0.71 (0.70–0.72)	< 0.001	0.72 (0.71–0.73)	< 0.001	0.74 (0.73–0.75)	< 0.001
SPISE (per 1 unit)	0.82 (0.82–0.83)		0.83 (0.83–0.84)		0.84 (0.84–0.85)	
T1 (Low)	1.00 (Reference)		1.00 (Reference)		1.00 (Reference)	
T2 (Middle)	0.70 (0.68–0.71)	< 0.001	0.72 (0.70–0.74)	< 0.001	0.73 (0.71–0.75)	< 0.001
T3 (High)	0.50 (0.48–0.51)	< 0.001	0.52 (0.51–0.54)	< 0.001	0.54 (0.52–0.55)	< 0.001
P for trend		< 0.001		< 0.001		< 0.001

P values were derived from Wald tests. All tests were two-sided

P for trend was calculated by modeling SPISE tertiles as an ordinal variable (coded 1, 2, 3) in the Cox models, testing for a linear trend across increasing tertiles

Model 1: Unadjusted, stratified by age groups (< 45, 45–59, ≥ 60 years) and sex

Model 2: Model 1 + ethnicity, education, Townsend index, smoking, alcohol, dietary score

Model 3: Model 2 + statin use, blood pressure medication, anti-diabetic medication (primary model)

**Fig. 3:**
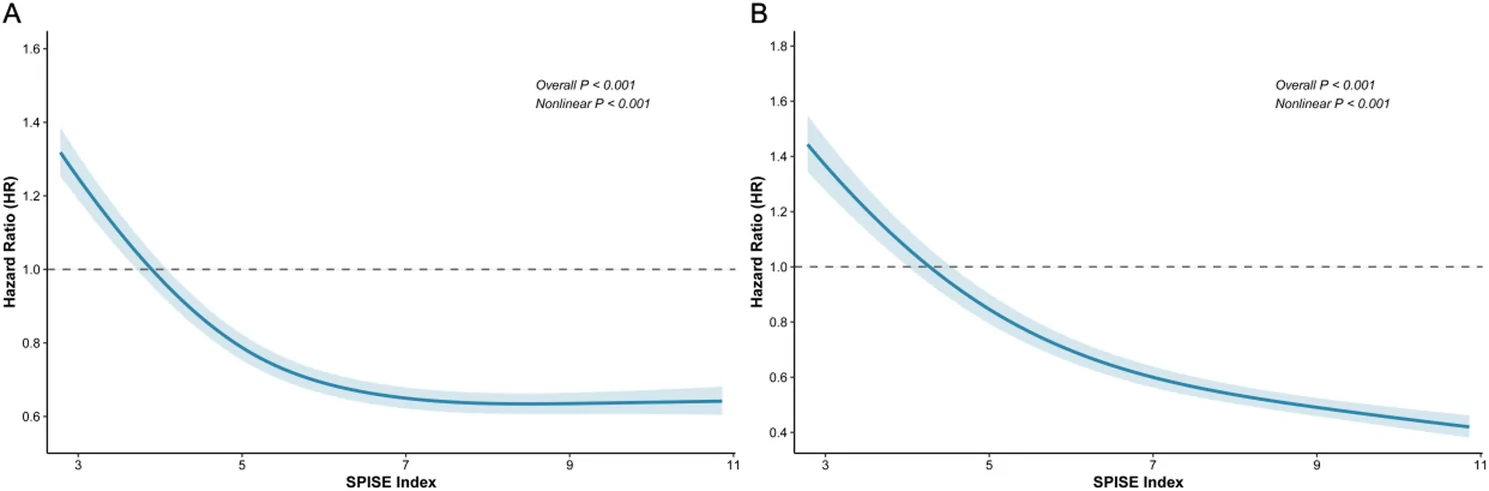
Restricted cubic spline analysis of the association between SPISE index and CVD (**A**) and CHD (**B**) risk. The overall association P values and P values for nonlinearity were calculated using Wald tests from the model ANOVA

Figure 3 presents the RCS analysis, systematically evaluating the dose-response relationship between SPISE and CVD incidence and CHD risk in individuals with CKM syndrome stages 0–3. Results reveal a significant inverse correlation between SPISE and both overall CVD risk and CHD event risk, with both exhibiting nonlinear associations. The inflection point for both correlations is 5.81 (nonlinear *P* < 0.001). In contrast, the relationship between SPISE and CVD risk follows an L-shaped pattern.

### Subgroup analyses

**Fig. 4:**
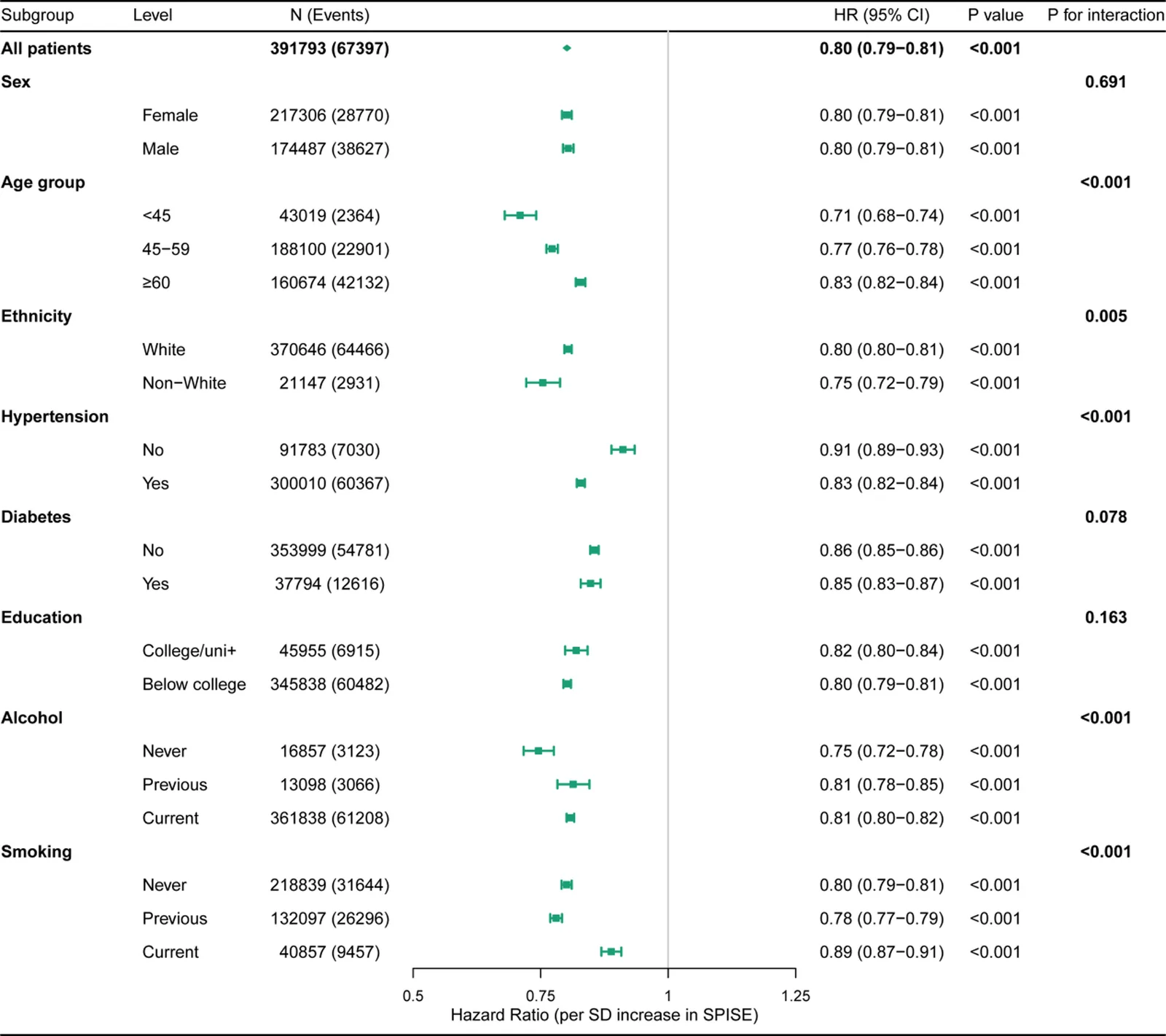
Subgroup analysis association of SPISE index and CVD incidence in CKM syndrome stage 0–3. P values for the subgroup-specific HRs were derived from Wald tests in the Cox models. P values for interaction were calculated using likelihood ratio tests comparing models with and without the interaction term between continuous SPISE and each subgroup variable

Subgroup analyses were conducted to assess whether pre-specified subgroups within the cohort modified the association between the SPISE index and CVD incidence. Subgroup analyses revealed heterogeneity in the SPISE-CVD association across certain subgroups (p for interaction < 0.005). The protective effect was most pronounced among younger participants (<45 years: HR 0.71; 95% CI 0.68–0.74), followed by non-white participants (HR 0.75; 95% CI 0.72–0.79). Similar results were observed for CHD (<45 years: HR 0.55; 95% CI 0.51–0.59), followed by non-white groups (HR 0.65; 95% CI 0.61–0.69). Furthermore, SPISE-CVD interaction effects were observed in subgroups based on hypertension and smoking status. No interaction effects were detected in subgroups based on other covariates (Fig. 4 and Figure S1). The effects associated with age and smoking status persist even when stratified by SPISE tertiles (Figure S2, S3).

To further validate the dose-gradient relationship between exposure and response, we re-stratified SPISE into quartiles and used the lowest quartile (Q1) as the reference. Results showed significantly higher CVD and CHD risks in Q2, Q3, and Q4 groups compared to Q1. HR (95% CI) values for CVD were 0.75 (0.73–0.76), 0.65 (0.64–0.67), and 0.59 (0.58–0.61), respectively. while CHD HRs were 0.72 (0.70–0.74), 0.58 (0.56–0.60), and 0.44 (0.43–0.46), respectively.This indicates a clear dose-response relationship, supporting the robustness of the negative association between exposure factors and outcomes. (Figure S4, S5)

### Sensitivity analyses

The primary findings remained robust across various sensitivity analyses. First, after further adjusting for insomnia status, comorbidities (diabetes, hypertension, hyperlipidemia), and CKM staging in Model 3, Cox proportional hazards model (Table S9) showed that the SPISE index continued to exhibit a significant negative association with the incidence of CVD and CHD risk. Second, after excluding participants who experienced CVD/CHD events within 3 years of follow-up (Table S10), a significant dose-response relationship between the SPISE index and overall CVD risk and CHD risk was still observed in the fully adjusted model. Subsequently, potential selection bias from participants with incomplete data was addressed (Table S11-14), and the results from the complete case analysis were highly consistent with our primary findings.

## Discussion

This study is the first to evaluate the impact of the SPISE index on cardiovascular disease risk in patients with stage 0–3 CKM syndrome. Key findings can be summarized as follows: [1] SPISE exhibits a significant negative correlation with overall CVD risk and CHD event risk, demonstrating a nonlinear relationship; [2] Cox proportional hazards models indicate that CVD risk progressively decreases with increasing SPISE tertiles, with a more pronounced reduction in CHD risk compared to CVD risk [3]. Cumulative risk curves across SPISE tertiles showed statistically significant differences (Log-rank *P* < 0.05), with higher SPISE levels corresponding to lower CVD risk [4]. Subgroup analysis revealed that the SPISE-CVD association exhibited a more pronounced protective effect in individuals under 45 years of age. In summary, SPISE exhibits a dose-dependent negative correlation with CVD risk in individuals with CKM stages 0–3, demonstrating significant protective effects.

SPISE is a biomarker that can estimate insulin sensitivity through routine biochemical markers, demonstrating significant predictive potential in cardiovascular diseases, metabolic disorders, and other conditions [15, 17, 20]. Research indicates that SPISE demonstrates superior predictive value for IR compared to QUICKI and HOMA-IR [13]. Subsequently, SPISE has demonstrated favorable clinical performance in multiple studies involving the diagnosis of Type 2 Diabetes Mellitus (T2DM), MASLD, HF, and CHD [21–23]. Clinical evidence indicates that a higher SPISE index is significantly associated with a reduced risk of future cardiovascular outcomes in patients with T2DM, with a HR of 0.85 (95% CI 0.79, 0.92) for cardiovascular mortality and 0.91 (95% CI 0.87, 0.95) for major coronary events [15]. Meanwhile, Cederholm J et al. reported that each standard deviation increase in SPISE also elevated the risk of fatal and non-fatal coronary heart disease (HR: 1.20; 95% CI 1.02, 1.40) [21]. A recent study further revealed that higher SPISE scores are significantly associated with reduced CVD risk [17]. In summary, SPISE demonstrates strong predictive value for IR, cardiovascular disease, and metabolic disorders, with a close association between SPISE and cardiovascular risk. Collectively, these findings suggest SPISE may serve as a valuable prognostic indicator for CKM syndrome.

Although the underlying mechanisms linking SPISE index to future CVD risk in patients with CKM syndrome stages 0–3 remain unclear, IR and metabolic alterations associated with these stages may contribute to this development. Research indicates that IR impacts lipid metabolism, particularly small dense low-density lipoprotein cholesterol (sdLDL-C)—a substance with potent atherogenic potential and an independent risk factor for CVD [24, 25]. Eor J et al. found in a cohort study of obese adults in South Korea that the SPISE index serves as an effective indicator for assessing the particle advantage of sdLDL-C [26]. That is, the metabolic abnormalities underlying a low SPISE index—including lower HDL cholesterol, elevated triglycerides, and higher BMI—may promote atherosclerosis and CVD by mediating lipid metabolism, inducing inflammation, enhancing endothelial injury, and activating the fibrinolytic system through sdLDL-C driven by insulin resistance [25]. Additionally, the SPISE index is calculated by integrating TG, HDL, and BMI levels. Elevated TG levels promote the formation of sdLDL-C [25]. Concurrently, HDL-c is recognized as a key factor in preventing atherosclerosis, and its reduction exacerbates atherosclerotic plaque formation [27]. The TG/HDL ratio is considered an effective marker for predicting CVD risk [28]. In contrast, the SPISE index incorporates BMI. Studies indicate that the SPISE index outperforms the TG/HDL ratio in diagnosing insulin sensitivity, insulin resistance, and MetS [15]. Multiple studies have demonstrated the significant role of BMI in the development of CVD [29, 30]. A prospective cohort study revealed that individuals with normal childhood BMI but elevated adult BMI exhibited a significantly higher risk of progressing to intermediate and advanced stages of CKD compared to those with normal BMI throughout childhood and adulthood. The odds ratio (OR) for advanced CKD was 5.78 (95% CI 3.74–8.93) [31]. Khan SS et al. also noted that obesity, compared to normal BMI, is associated with a significantly increased risk of cardiovascular disease and mortality [32]. In this study, we obtained similar findings: individuals with high SPISE levels exhibited elevated HDL-C, lower TG, and lower BMI. These individuals demonstrated reduced risks of CVD and CHD. Potential reasons include: First, obesity-induced hemodynamic alterations may directly induce changes in cardiovascular structure and function, such as left ventricular morphological abnormalities; Second, scholars have noted that adipokine dysregulation serves as a central hub in obesity-induced CVD. Imbalances in adipokines lead to the release of pro-inflammatory components like leptin and interleukin-6, which promote atherosclerosis. Third, this relates to regional fat distribution and abnormal fat accumulation. Visceral fat, in particular, exhibits strong associations with insulin resistance, atherosclerotic dyslipidemia, hypertension, and CVD risk [29, 33]. In summary, the SPISE index may be regarded as a reflection of metabolic imbalance that plays a significant role in cardiovascular event occurrence among patients with CKM syndrome stages 0–3. Although these mechanisms remain under-validated in the CKM population, their convergence across studies provides critical insights into the multidimensional pathophysiological mechanisms underlying the SPISE index’s association with cardiovascular outcomes in this cohort.

Furthermore, this pathophysiological complexity manifests as clinical heterogeneity. Subgroup analyses revealed that younger individuals under 45 years of age, as well as former smokers and never-smokers, demonstrated greater potential benefits in both overall CVD risk and coronary heart disease risk assessments, potentially attributable to metabolic differences. A previous cohort study from the UKB also revealed that aging is associated with increased inflammation, impaired glucose homeostasis, and elevated blood pressure [34, 35]. The concept of “metabolic plasticity” proposed by Zhou Q et al.—defined as the ability to switch to an adaptive metabolic state during changes in nutrient availability and then revert to a pre-established state when nutritional conditions normalize—provides a mechanistic explanation for this phenomenon. Aging represents a particularly significant contextual factor contributing to the decline in this plasticity [36]. Based on this, it can be inferred that individuals with higher metabolic resilience (such as the previously mentioned < 45-year-olds and former smokers versus never-smokers) exhibit greater metabolic adaptability to interventions, thereby demonstrating more pronounced benefits. Conversely, the age-related decline in resilience may diminish the responsiveness of older populations to equivalent interventions. Regarding smoking, epidemiological studies indicate that it is associated with multiple cardiovascular diseases, such as fatal CHD and myocardial infarction [37, 38]. Furthermore, smoking is a major risk factor for metabolic disorders, particularly type 2 diabetes. Research indicates that cigarette smoke contains thousands of active components, whose combined exposure can disrupt metabolic regulatory networks by targeting key inflammatory genes [39]. These findings advocate for personalized cardiovascular disease prevention strategies based on SPISE, tailored to age and lifestyle habits, thereby laying a solid foundation for precision medicine.

The primary strength of this study lies in its pioneering exploration, to our knowledge, of the predictive efficacy of the SPISE index for future CVD risk in patients with CKM stages 0–3. Furthermore, this assessment is grounded in a large sample size and comprehensive long-term follow-up data, yielding objective and reliable results. Additionally, the SPISE index requires only a single fasting blood sample, making it a simple test with practical potential for screening high-risk cardiovascular individuals in large-scale populations.

Our study also has several limitations. First, SPISE was assessed only at baseline, and metabolic status may change substantially over time. Therefore, although TG, HDL, and BMI levels were meticulously recorded at various follow-up time points, we were unable to calculate the impact of SPISE index variability on future cardiovascular outcomes. This limitation may underestimate the long-term cardiovascular risk associated with metabolic fluctuations. Second, despite rigorous covariate adjustments and sensitivity analyses, unmeasured or residual confounding factors remain and cannot be disregarded. Third, as this study is based on a UK population, caution is warranted when extrapolating conclusions to other ethnic groups (e.g., Asian and African populations), and further validation across diverse populations is needed. For example, in a South African Black community, triglyceride-dependent insulin resistance indices (e.g., TyG) failed to associate with end-organ damage, whereas HOMA-IR and QUICKI remained effective [40]. Fourth, physical activity was assessed using self-reported questionnaires, which are subject to recall bias and social desirability bias. The UK Biobank is known to have a ‘healthy volunteer’ selection bias, and the definition of adequate physical activity (≥ 150 min/week of walking or moderate-intensity activity, or ≥ 75 min/week of vigorous-intensity activity) included walking as a qualifying activity. These factors may partially explain the unexpectedly high proportion of participants meeting physical activity guidelines, even among those with obesity (mean BMI 32 kg/m²) in the lowest SPISE tertile. Fifth, our study was not designed to quantify the incremental prognostic value of SPISE beyond its individual components (HDL-C, TG, BMI) or established risk scores. Future studies specifically designed to evaluate this incremental predictive value using appropriate metrics (e.g., C-statistic, NRI, IDI) are warranted to further establish the clinical utility of the SPISE index.

## Conclusion

This large prospective cohort study demonstrates that elevated SPISE levels are significantly associated with reduced risks of first cardiovascular events and coronary heart disease in patients with stages 0–3 CKM syndrome. The SPISE indicator is not only cost-effective but also holds potential clinical value, showcasing its promise as a novel monitoring biomarker in the clinical management of CKM syndrome.

## Supplementary Information

Below is the link to the electronic supplementary material.

Supplementary Material 1

## Data Availability

Research conducted using the UK Biobank Resource under Application number 220028. The UK Biobank resource can be accessed by researchers on application. Variables derived for this study will be returned to the UK Biobank for future applicants to request. No additional data are available.
