# Supplementary material for: Association of the single-point insulin sensitivity estimator (SPISE) index and incident cardiovascular disease in early-stage CKM syndrome: insights from the UK biobank
Source: Diabetol Metab Syndr. 2026 Jun 25;18:193. doi: 10.1186/s13098-026-02222-z (PMC13560290; doi:10.1186/s13098-026-02222-z)
Supplement: Supplementary file 1 [file 13098_2026_2222_MOESM1_ESM.docx]

**Supplementary Document**

**Association of the Single-Point Insulin Sensitivity Estimator (SPISE) Index with Cardiovascular Events Among Patients with Early-Stage CKM Syndrome: Evidence from the UK Biobank Cohort**

**Table of contents**

**Table S1**. Assessment of CKM Syndrome Stages

**Table S2**. ICD codes and Field ID defining diseases in UKB

**Table S3**. Definition and assessment of variables

**Table S4**. Drug Classification and Data Coding

**Table S5**. The scoring system of diet

**Table S6**. Variance Inflation Factors for Covariates in Models of CVD and CHD by SPISE Tertiles and Continuous SPISE

**Table S7**. The Akaike information criterion and the Bayesian information criterion in model selection

Table S8. Baseline Characteristics by CKM Stages

**Table S9**. The performance of the SPISE index in Model 4 with CVD and CHD as the outcome

**Table S10**. The performance of the SPISE index with CVD and CHD as the outcome after excluding events occurring within the first three years of follow-up

**Table S11**. The performance of the SPISE tertiles with CVD as the outcome after excluding participants with incomplete Data

**Table S12**. The performance of the SPISE tertiles with CHD as the outcome after excluding participants with incomplete Data

**Table S13**. The performance of the Continuous SPISE with CVD as the outcome after excluding participants with incomplete Data

**Table S14**. The performance of the Continuous SPISE with CHD as the outcome after excluding participants with incomplete Data

**Table S15**. Simplified PREVENT model for 10 year CVD

**Table S16**. CKD-EPI equations for estimating GFR on the natural scale expressed for specified sex, serum creatinine or serum cystatin C

**Figure of contents**

**Figure S1.** Analysis association of SPISE index and CHD incidencec in CKM syndrome stage 0–3

**Figure S2.** Analysis association of SPISE tertiles and CVD incidencec in CKM syndrome stage 0–3

**Figure S3.** Analysis association of SPISE tertiles and CHD incidencec in CKM syndrome stage 0–3

**Figure S4.** Analysis association of SPISE quartiles and CVD incidencec in CKM syndrome stage 0–3

**Figure S5.** Analysis association of SPISE quartiles and CHD incidencec in CKM syndrome stage 0–3

**Table S1. Assessment of CKM Syndrome Stages**

Based on variables available in the UKB database, the CKM staging system was adjusted in accordance with the methodology previously described by Zhang et al. [18]. Age, sex, race, and ethnicity were selfreported. Comorbidities (e.g., diabetes, hypertension, dyslipidemia) and cardiovascular disease (CVD) history(including coronary heart disease, stroke, atrial fibrillation, heart failure, and peripheral artery disease) were ascertained through primary care records, hospitalization data, death registries, and self-reported medical conditions. Estimated glomerular filtration rate (GFR) was calculated using the race-free CKD-EPI 2021 creatinine equation.CKM Stage 0 was defined as individuals with normal body mass index (BMI; <23 kg/m² for Asian ancestry and <25kg/m² for other racial/ethnic groups) and normal waist circumference (<80 cm for Asian women and <90 cm for Asian men; <88 cm for women and <102 cm for men of other ancestries), without meeting criteria for advanced stages.

| **CKM syndrome stages** | **Definition** |
| --- | --- |
| Stage 0: No CKM risk factors | Individuals with normal body mass index (BMI; <23 kg/m² for Asian ancestry and <25 kg/m² for other racial/ethnic groups) and normal waist circumference (<80 cm for Asian women and <90 cm for Asian men; <88 cm for women and <102 cm for men of other ancestries), without meeting criteria for advanced stages. |
| Stage 1: Excess or dysfunctional adiposity | Individuals with overweight/obesity, abdominal obesity, or dysfunctional adipose tissue, characterized by elevated BMI (≥23 kg/m² for Asian ancestry and ≥25 kg/m² for other groups), elevated waist circumference (≥80 cm for Asian women and ≥90 cm for Asian men; ≥88 cm for women and ≥102 cm for men of other ancestries), or prediabetes (glycated hemoglobin 5.7% to <6.5% or fasting blood glucose 100 mg/dL to <126 mg/dL), in the absence of additional metabolic risk factors or chronic kidney disease (CKD). |
| Stage 2: Metabolic risk factors and CKD | Individuals with metabolic risk factors or moderate-to-high-risk CKD per Kidney Disease Improving Global Outcomes (KDIGO) criteria. Metabolic risk factors included elevated fasting triglycerides (≥135 mg/dL), hypertension, diabetes, or metabolic syndrome (defined as ≥3 of the following: waist circumference ≥88 cm for women and ≥102 cm for men (≥80 cm for women and ≥90 cm for men of Asian ancestry); HDL cholesterol <40 mg/dL for men and <50 mg/dL for women; triglycerides ≥150 mg/dL; elevated blood pressure (systolic blood pressure ≥130 mm Hg, diastolic blood pressure ≥80 mm Hg, or antihypertensive medication use), self-reported hypertension, and ICD-10 diagnosed hypertension. ); or fasting glucose ≥100 mg/dL. Participants with dyslipidemia were also classified into this stage. Due to substantial missing data for urinary albumin-creatinine ratio (available in 38.9% of the cohort), CKD staging primarily relied on eGFR <60 mL/min/1.73 m². |
| Stage 3: Subclinical CVD in CKM | By the presence of very-high-risk CKD per KDIGO criteria (GFR categories 4-5 or KDIGO heat map risk stratification) or a 10-year predicted CVD risk ≥20% using the AHA PREVENT equations. The PREVENT equations were applied to adults aged 30-79 years, with participants ≥80 years conservatively assigned an age of 79 years for risk estimation. Variables outside PREVENT-defined ranges (total cholesterol: 130-320 mg/dL; HDL: 20-100 mg/dL; systolic blood pressure: 90-200 mmHg; eGFR: 14-140 mL/min/1.73 m²) were truncated to boundary values. |
| Stage 4: Clinical CVD in CKM | Individuals with established CVD (coronary heart disease, stroke, atrial fibrillation, heart failure, or peripheral artery disease) documented through primary care records, hospitalization data, death registries, or self-reported diagnoses, occurring in the context of excess/dysfunctional adiposity, CKM risk factors, or CKD. |

**Table S2. ICD codes and Field ID defining diseases in UKB**

First-onset cardiovascular disease (CVD), including coronary heart disease (CHD), stroke, atrial fibrillation (AF), heart failure (HF), and peripheral arterial disease (PAD), was ascertained from the UK Biobank (UKB) "first occurrence" dataset (Category 1712). This dataset integrates diagnoses recorded before and after the baseline assessment visit, identified through linkage to primary care records, hospitalization data, death registries, and self-reported medical conditions. Disease-specific International Classification of Diseases, Tenth Revision (ICD-10) three-character codes were mapped as follows: I20.0–I25.9 for CHD, I60.0–I64 for stroke, I48–I48.9 for AF, I50.0–I50.9 for HF, and I70.0–I79.8 for PAD.

For each participant, the earliest recorded diagnosis date across all data sources was assigned as the incident date. During the baseline assessment, trained nurses collected self-reported medical histories by asking participants whether they had ever received a physician-confirmed diagnosis of specific conditions. When both registry-derived ICD-10 codes (from primary care, hospitalizations, or death records) and self-reported diagnoses were available, the earliest date between the two sources was prioritized. Further details on the derivation of "first occurrence" data are available in the UK Biobank documentation (https://biobank.ndph.ox.ac.uk/showcase).

| **Disease** | **Data field ID** | **ICD-10** |
| --- | --- | --- |
| Cardiovascular disease |  |  |
| Coronary heart disease | 131296; 131298; | I20.0;I20.1;I20.8;I20.9; I21.0; I21.1; I21.2; I21.3; I21.4; I21.9; I22.0; I22.1; |
|  | 131300; 131302; | I22.8; I22.9; I23; I23.0; I23.1; I23.2; I23.3; I23.5; I23.6; I23.8; I24.0; I24.1; |
|  | 131304; 131306 | I24.8; I24.9; I25.0; I25.1; I25.2; I25.3; I25.4; I25.5; I25.6; I25.8; I25.9; |
| Stroke | 131360; 131362; | I60.0; I60.1; I60.2; I60.3; I60.4; I60.5; I60.6; I60.7; I60.8; I60.9; I61.0; I61.1; |
|  | 131364; 131366; 131368 | I61.2; I61.3; I61.4; I61.5; I61.6; I61.8; I61.9; I62.1; I61.9; I63.0; I63.1; I63.2; |
|  |  | I63.3; I63.4; I63.5; I63.6; I63.8; I63.9; I64; |
| Heart failure | 131354 | I50.0; I50.1; I50.9; |
| Atrial fibrillation | 131350 | I48.0; I48.1; I48.2; I48.3; I48.4; I48.9 |
| PAD | 131370; 131372; | I70.0; I70.00; I70.01; I70.1; I70.10; I70.11; I70.2; I70.20; I70.21; I70.8; I70.80; |
|  | 131374; 131376; | I70.81; I70.9; I70.90; I70.91; I71.0; I71.1; I71.2; I71.3; I71.4; I71.5; I71.6; |
|  | 131378; 131380; | I71.8; I71.9; I72.0; I72.1; I72.2; I72.3; I72.4; I72.5; I72.6; I72.8; I72.9; I73.0; |
|  | 131382; 131384; | I73.1; I73.8; I73.9; I74.0; I74.1; I74.2; I74.3; I74.4; I74.5; I74.8; I74.9; I77.1; |
|  | 131386; 131388; | I77.2; I77.3; I77.4; I77.5; I77.6; I77.8; I77.9; I78.0; I78.1; I78.8; I78.9; I79.1; |
|  | 131390; 131392; 131394 | I79.2; I79.8 |
| Type 2 diabetes | 130708 | E11.0; E11.1; E11.2; E11.3; E11.4; E11.5; E11.6; E11.7; E11.8; E11.9; |
| Hypertension | 131286; 131288; | I10; I11.0; I11.9; I12.0; I12.9; I13.0; I13.1; I13.2 I13.9; I15.0; I15.1; I15.2; |
|  | 131290; 131292; | I15.8; I15.9 |
|  | 131294; |  |
| Dyslipidemia | 130814 | E78.0; E78.1; E78.2; E78.3; E78.4; E78.5; E78.6; E78.8; E78.9; |

Note: ICD: international classification diseas

**Table S3. Definition and assessment of variables**

| **Variables** | **Defintion** | **Assessment** | **UK Biobank Data-Field ID** |
| --- | --- | --- | --- |
| Age(years) | Age in years. | Interval between the date of baseline assessment and the date of birth recorded by NHS. | 21003 |
| Sex | Male, Female. | NHS derived and/or touchscreen questionnaire. | 31 |
| Ethnic background | White, Non-White | Touchscreen questionnaire. | 21000 |
| Education | Less than high school，High school or equivalent，College or above | Touchscreen questionnaire: “Which of the following qualifications do you have?” | 6138 |
| Alcohol consumption status | Never, Previous, Current. | Touchscreen questionnaire: “Did you previously drink alcohol?” and “About how often do you drink  alcohol?” | 20117 |
| Smoking status | Never, Previous, Current. | Touchscreen questionnaire: “Do you smoke tobacco now?” and “In the past, how often have you smoked tobacco?” | 20116 |
| Insomnia | Never/rarely, Sometimes, Usually | Touchscreen questionnaire: “Do you have trouble falling asleep at night or do you wake up in the middle of the night?” | 1200 |
| Dietary Score | See supplementary table S5 for details | See supplementary table S5 for details | 1289, 1299,1309, 1329, 1339,1349, 1359, 1369,1379, 1389 |
| Townsend deprivation index | Townsend deprivation index calculated immediately prior to participant joining UK Biobank. | Townsend Deprivation Index is a composite score of four aspects (unemployment, non-car ownership, nonhome ownership, and household overcrowding), which was obtained from the residents’ postcodes using census information. | 189 |
| Physical activity | Above recommendation，  Not meeting the  recommendation | Touchscreen questionnaire: “In a typical WEEK, on how many days did you do 10 minutes or more of moderate physical activities like carrying light loads, cycling at normal pace? (Do not include walking).In a typical WEEK, how many days did you do 10 minutes or more of vigorous physical activity? (These are activities that make you sweat or breathe hard such as fast cycling, aerobics, heavy lifting). In a typical WEEK, on how many days did you walk for at least 10 minutes at a time? (Include walking that you do at work, travelling to and from work, and for sport or leisure). How many minutes did you usually spend doing moderate activities on a typical DAY? How many minutes did you usually spend doing vigorous activities on a typical DAY? How many minutes did you usually spend walking on a typical DAY?” | 894, 914, 874, 884, 904, 864 |
| Waist circumference | Units of measurement are cm | Waist circumference here is measured during the initial Assessment Centre visit | 48 |
| Body mass index | Body Mass Index. Units of measurement are Kg/m2 | BMI value here is constructed from height and weight measured during the initial Assessment Centre visit. Value is not present if either of these readings were omitted | 21001 |
| HDL-C | Units of measurement are mmol/L | Measured by enzyme Immuno-inhibition analysis on a Beckman Coulter AU5800. | 30760 |
| Triglyceride | Units of measurement are mmol/L | Measured by enzymatic analysis on a Beckman Coulter AU5800 | 30870 |
| Cholesterol | Units of measurement are mmol/L. | Measured by enzymatic analysis on a Beckman Coulter AU5800 | 30690 |
| Fasting Blood Glucose | Units of measurement are mmol/L. | Measured by hexokinase analysis on a Beckman Coulter AU5800 | 30740 |
| HbA1c | Units of measurement are mmol/mol | Measured by HPLC analysis on a Bio-Rad VARIANT II Turbo | 30750 |
| C reactive protein | Units of measurement are mg/L. | Measured by enzyme Immuno-inhibition analysis on a Beckman Coulter AU5800. | 30710 |
| Creatinine (enzymatic) in urine | Units of measurement are mmol/L. | Measured by enzymatic analysis on a Beckman Coulter AU5400. | 30510 |
| Albumin | Units of measurement are μmol/L. | Measured by BCG analysis on a Beckman Coulter AU5800 | 30600 |
| Creatinine | Units of measurement are μmol/L. | Measured by enzymatic analysis on a Beckman Coulter AU5800 | 30700 |
| Cystatin C | Units of measurement are mg/L. | Measured by latex enhanced immunoturbidimetric analysis on a Siemens ADVIA 1800 | 30720 |
| Systolic blood pressure | Units of measurement are mmHg. | Blood pressure, automated reading or manual reading, systolic. Two measures of blood pressure were taken a few moments apart | 4080, 93 |
| Diastolic blood pressure | Units of measurement are mmHg. | Blood pressure, automated reading or manual reading, diastolic. Two measures of blood pressure were taken a few moments apart | 4079, 94 |

*Physical activity that met the 2017 UK Physical activity guidelines (150 min of walking or moderate activity per week or 75 min of vigorous activity) was defined as above recommendation.

HbA1c, Glycated Hemoglobin A1c; HDL, High-Density Lipoprotein; IR, Insulin Resistance; TyG, Triglyceride-Glucose Index; FBG, Fasting Blood Glucose; TyG-BMI, Triglyceride-Glucose-Body Mass Index; AIP, Atherogenic Index of Plasma; TyG-WC, Triglyceride-Glucose-Waist Circumference; BMI, Body Mass Index; WC, Waist Circumference; SD, Standard Deviation; CVD, Cardiovascular Disease; CKD, Chronic Kidney Disease; CKM, Cardiovascular-Kidney-Metabolic.

**Table S4. Drug Classification and Data Coding**

| 1. Lipid-lowering medication | | 20003;6177;6153 |
| --- | --- | --- |
| 1140861922; 1141146234; 1140910654; 1141192410; 1140861958; 1140888594; 1140888648; 1140861970; 1140910632; 140861946;1140862028; 1141192414; 1140861924; 1140861954; 1141157260; | | |
| 1140861944; 1140861856; 1141157262; 1140861926; 1140861928; 1140861936; 1140861942; 140861858;1140888590;1140910652;1140861868;1140861892;1140861876; 1140861866; 1140861848; | | |
| 1140861878;1140861894; 1140865576;1140909780; 1141157416; | | |
| 2. Antihypertensive medication | | 20003;6177;6153 |
| 1140851692; 1140860696; 1140860706; 1140860714; 1140860728; 1140860736; 1140860750; 1140860752; 1140860758; 1140860764; 1140860776; 1140860784; 1140860790; 1140860802; 1140860806; | | |
| 1140860878; 1140860882; 1140860892; 1140860904; 1140860912; 1140860918; 1140864618; 1140864910; 1140864952; 1140866078; 1140879802; 1140881706; 1140888552; 1140888556; 1140888560; | | |
| 1140888646; 1140916356; 1140916362; 1140923718; 1141145660; 1141145668; 1141150328; 1141150560; 1141151016; 1141151018; 1141152998; 1141153006; 1141153316; 1141153328; 1141156836; | | |
| 1141156846; 1141164148; 1141164154; 1141165470; 1141165476; 1141166006; 1141167758; 1141167822; 1141170544; 1141170870; 1141171336; 1141172492; 1141172682; 1141172686; 1141179974; | | |
| 1141180592; 1141180598; 1141187788; 1141188408; 1141190934; 1141193282; 1141193346; 1141199940; 1141200698; 1141200726; 1141201038; 1141201040; 1140851332; 1140851336; 1140851338; | | |
| 1140851362; 1140851364; 1140851368; 1140851660; 1140860332; 1140860404; 1140860422; 1140860562; 1140860738; 1140864950; 1140866072; 1140866074; 140866084; 1140866086; 1140866092; | | |
| 1140866096; 1140866108; 1140866110; 1140866128; 1140866132; 1140866136; 1140866138; 1140866140; 1140866146; 1140866156; 1140866158; 1140866162; 1140866164; 1140866168; 1140866422; | | |
| 1140866440; 1140866446; 1140888918; 1140888922; 1140909706; 1140916870; 1140917068; 1140926778; 1141146378; 1141180772; 1141180778; 1141188636; 1141194794; 1141194800; 1141194804; | | |
| 1141194808; 1141194810; 1140851360; 1140851418; 1140851420; 1140851428; 1140851430; 1140851436; 1140860292; 1140860322; 1140860336; 1140864550; 1140864574; 1140866220; 1140866226; | | |
| 1140866232; 1140866236; 1140866244; 1140866248; 1140866280; 1140866282; 1140866306; 1140866308; 1140866312; 1140866318; 1140866324; 1140866328; 1140866330; 1140866332; 1140866334; | | |
| 1140866340; 1140866352; 1140866354; 1140866356; 1140866360; 1140866388; 1140866390; 1140866396; 1140866400; 1140866402; 1140866404; 1140866406; 1140866410; 1140866416; 1140866418; | | |
| 1140866420; 1140866426; 1140866438; 1140866442; 1140866444; 1140866448; 1140881894; 1140888512; 1140909708; 1140909722; 1140922324; 1140923272; 1140923276; 1140923282; 1140923402; | | |
| 1140927174; 1140928624; 1141146128; 1141150898; 1141167108; 1141181520; 1141195254; 1141195258; 1141201250; 1140851508; 1140851522; 1140851556; 1140860172; 1140860180; 1140860232; | | |
| 1140860250; 1140860266; 1140860278; 1140860308; 1140860314; 1140860318; 1140860320; 1140860324; 1140860328; 1140860330; 1140860338; 1140860340; 1140860342; 1140860348; 1140860356; | | |
| 1140860358; 1140860386; 1140860394; 1140860396; 1140860398; 1140860402; 1140860406; 1140860410; 1140860426; 1140860434; 1140860492; 1140860498; 1140861090; 1140864176; 1140864410; | | |
| 1140866704; 1140866712; 1140866724; 1140866738; 1140866756; 1140866758; 1140866764; 1140866766; 1140866778; 1140866782; 1140866784; 1140866798; 1140866800; 1140866802; 1140866804; | | |
| 1140867734; 1140879758; 1140879760; 1140879762; 1140879818; 1140879824; 1140879834; 1140879854; 1140879866; 1140881702; 1140881722; 1140909368; 1140916628; 1140916730; 1140916868; | | |
| 1140917076; 1140922930; 1140923336; 1141146124; 1141146126; 1141146184; 1141152076; 1141156754; 1141164280; 1141168498; 1141169516; 1141171152; 1141172742; 1141182904; 1141182968; | | |
| 1141184324; 1141184722; 1141187048; 1141187780; 1140851730; 1140851784; 1140851786; 1140851790; 1140851800; 1140855976; 1140861088; 1140861106; 1140861110; 1140861114; 1140861120; | | |
| 1140861128; 1140861130; 1140861136; 1140861138; 1140861176; 1140861190; 1140861202; 1140861276; 1140861282; 1140866460; 1140866466; 1140866484; 1140866546; 1140866554; 1140868036; | | |
| 1140872472; 1140872568; 1140879810; 1140881692; 1140888510; 1140911088; 1140911698; 1140916930; 1140917428; 1140923572; 1140923618; 1140926188; 1140926780; 1140927934; 1140927940; | | |
| 1140928212; 1140928226; 1140928234; 1141145870; 1141150500; 1141150538; 1141150926; 1141151474; 1141152600; 1141153026; 1141153032; 1141153394; 1141153454; 1141156656; 1141157136; | | |
| 1141157140; 1141162546; 1141166752; 1141167832; 1141169096; 1141169710; 1141169730; 1141171804; 1141173766; 1141174684; 1141175224; 1141180238; 1141184390; 1141185444; 1141187056; | | |
| 1141187094; 1141187774; 1141187962; 1141188152; 1141188576; 1141188936; 1141190548; 1141199858; 1141200400; 1141200782; 1141201814 | | |
| 3. Antidiabetic medication | 20003; 6177; 6153 | |
| 1140857494; 1140874724; 1140857496; 1140874726;1140857500;1140874728; 1140857502; 1140874732; 1140857506; 1140874736; 1140874646; 1140874744; 1140874650; 1140874746; 1140874652; | | |
| 1140883066; 1140874664; 1140884600; 1140874666; 1141152590; 1140874674; 1141153254; 1140874678; 1141153262; 1140874680; 1141156984; 1140874686; 1141171646; 1140874690; 1141171652; | | |
| 1140874706; 1141177600; 1140874712; 1141177606; 1140874716; 1141189090; 1140874718; 1141189094; | | |

**Table S5. The scoring system of diet**

| Characteristics | Data field ID | Questions | Target Value |
| --- | --- | --- | --- |
| Fruit and Vegetables | 1289 | 1. On average how many heaped tablespoons of COOKED vegetables would you eat per DAY? | Sum ≥ 4.5 pieces or servings /day, |
|  | 1299 | 2. On average how many heaped tablespoons of SALAD or RAW vegetables would you eat per DAY? | Yes (1) / No (0) |
|  | 1309 | 3. About how many pieces of FRESH fruit would you eat per DAY? | 3 tablespoons of vegetables were |
|  |  |  | considered one serving |
| Fish | 1329 | 4. How often do you eat oily fish? (e.g. sardines, salmon, mackerel, herring) | Sum ≥2 intake/week, |
|  | 1339 | 5. How often do you eat other types of fish? (e.g. cod, tinned tuna, haddock) | Yes (1) / No (0) |
| Processed Meat | 1349 | 6. How often do you eat processed meats (such as bacon, ham, sausages, meat pies, kebabs, burgers, | ≤ 2 times/week intake of |
|  |  | chicken nuggets) | processed meat |
| Red Meat | 1369 | 7. How often do you eat beef? (Do not count processed meats) | and ≤ 5 times/week of red meat |
|  | 1379 | 8. How often do you eat lamb/mutton? (Do not count processed meats) | Intake, |
|  | 1389 | 9. How often do you eat pork? (Do not count processed meats such as bacon or ham) | Yes (1) / No (0) |

**Table S6. Variance Inflation Factors for Covariates in Models of CVD and CHD by SPISE Tertiles and Continuous SPISE**

| **Variable** | **Continous** | | **Spise tertile** | |
| --- | --- | --- | --- | --- |
|  | **VIF** | **Collinearity_Level** | **VIF** | **Collinearity_Level** |
| **CVD** |  |  |  |  |
| ethnicity_binary | 1.11 | no significant multicollinearity | 1.11 | no significant multicollinearity |
| sex | 1.10 | no significant multicollinearity | 1.11 | no significant multicollinearity |
| alcohol_cat | 1.10 | no significant multicollinearity | 1.09 | no significant multicollinearity |
| townsend | 1.09 | no significant multicollinearity | 1.09 | no significant multicollinearity |
| smoking_cat | 1.09 | no significant multicollinearity | 1.09 | no significant multicollinearity |
| SPISE | 1.07 | no significant multicollinearity | 1.07 | no significant multicollinearity |
| dietary_score_total | 1.07 | no significant multicollinearity | 1.07 | no significant multicollinearity |
| age_strata | 1.06 | no significant multicollinearity | 1.07 | no significant multicollinearity |
| bp_medication_for_ckm | 1.04 | no significant multicollinearity | 1.04 | no significant multicollinearity |
| education_binary | 1.02 | no significant multicollinearity | 1.02 | no significant multicollinearity |
| statin_use_for_ckm | 1.01 | no significant multicollinearity | 1.01 | no significant multicollinearity |
| anti_diabetic_med | 1.01 | no significant multicollinearity | 1.01 | no significant multicollinearity |
| **CHD** |  |  |  |  |
| ethnicity_binary | 1.13 | no significant multicollinearity | 1.13 | no significant multicollinearity |
| alcohol_cat | 1.11 | no significant multicollinearity | 1.11 | no significant multicollinearity |
| townsend | 1.10 | no significant multicollinearity | 1.10 | no significant multicollinearity |
| smoking_cat | 1.10 | no significant multicollinearity | 1.10 | no significant multicollinearity |
| sex | 1.09 | no significant multicollinearity | 1.10 | no significant multicollinearity |
| age_strata | 1.07 | no significant multicollinearity | 1.07 | no significant multicollinearity |
| dietary_score_total | 1.07 | no significant multicollinearity | 1.07 | no significant multicollinearity |
| SPISE | 1.06 | no significant multicollinearity | 1.06 | no significant multicollinearity |
| bp_medication_for_ckm | 1.04 | no significant multicollinearity | 1.04 | no significant multicollinearity |
| education_binary | 1.02 | no significant multicollinearity | 1.02 | no significant multicollinearity |
| statin_use_for_ckm | 1.01 | no significant multicollinearity | 1.01 | no significant multicollinearity |
| anti_diabetic_med | 1.01 | no significant multicollinearity | 1.01 | no significant multicollinearity |

**Table S7. The Akaike information criterion and the Bayesian information criterion in model selection**

|  | **SPISE Tertiles** | | | **Continuous SPISE** | | |
| --- | --- | --- | --- | --- | --- | --- |
|  | **Model1** | **Model2** | **Model3** | **Model1** | **Model2** | **Model3** |
| **CVD** |  |  |  |  |  |  |
| AIC | 1497199.3 | 1494115.0 | 1493291.7 | 1496891.2 | 1493841.4 | 1493060.4 |
| BIC | 1497217.6 | 1494206.2 | 1493410.3 | 1496900.3 | 1493923.5 | 1493169.8 |
| **CHD** |  |  |  |  |  |  |
| AIC | 716492.5 | 714815.3 | 714445.7 | 715979.7 | 714358.1 | 714030.7 |
| BIC | 716509.3 | 714899.1 | 714554.6 | 715988.1 | 714433.5 | 714131.3 |

Table S8. Baseline Characteristics by CKM Stages

| **Characteristic** | **Overall** (N = 391793)^1^ | **Stage 0** (N = 23235)^1^ | **Stage 1** (N = 16078)^1^ | **Stage 2** (N = 342398)^1^ | **Stage 3** (N = 10082)^1^ | **P value**^2^ |
| --- | --- | --- | --- | --- | --- | --- |
| **Age, years** | 56.1 (8.1) | 50.4 (7.3) | 51.4 (7.6) | 56.5 (7.9) | 64.3 (4.7) | <0.001 |
| **Sex** |  |  |  |  |  | <0.001 |
| Female | 217,306 (55%) | 17,692 (76%) | 11,162 (69%) | 185,856 (54%) | 2,596 (26%) |  |
| Male | 174,487 (45%) | 5,543 (24%) | 4916(31%) | 156,542 (46%) | 7,486 (74%) |  |
| **Ethnicity** |  |  |  |  |  | <0.001 |
| White | 370,646 (95%) | 22,268 (96%) | 14,881 (93%) | 324,111 (95%) | 9,386 (93%) |  |
| Non-White | 21,147 (5%) | 967 (4%) | 1,197 (7%) | 18,287 (5%) | 696 (7%) |  |
| **Education level** |  |  |  |  |  | <0.001 |
| College/uni+ | 45,955 (12%) | 3,654 (16%) | 2,089 (13%) | 39,359 (11%) | 853 (8%) |  |
| Below college | 345,838 (88%) | 19,581 (84%) | 13,989 (87%) | 303,039 (89%) | 9,229 (92%) |  |
| **Townsend deprivation index** | -1.4 (3.1) | -1.5 (3.0) | -1.3 (3.1) | -1.4 (3.0) | -0.8 (3.3) | <0.001 |
| **Smoking status** |  |  |  |  |  | <0.001 |
| Never | 218,839 (56%) | 14,744 (63%) | 9,534 (59%) | 191,179 (56%) | 3,382 (34%) |  |
| Previous | 132,097 (34%) | 6,399 (28%) | 5,007 (31%) | 116,958 (34%) | 3,733 (37%) |  |
| Current | 40,857 (10%) | 2,092 (9%) | 1,537 (10%) | 34,261 (10%) | 2,967 (29%) |  |
| **Alcohol consumption** |  |  |  |  |  | <0.001 |
| Never | 16,857 (4%) | 736 (3%) | 659 (4%) | 14,882 (4%) | 580 (6%) |  |
| Previous | 13,098 (3%) | 637 (3%) | 461 (3%) | 11,448 (3%) | 552 (5%) |  |
| Current | 361,838 (92%) | 21,862 (94%) | 14,958 (93%) | 316,068 (92%) | 8,950 (89%) |  |
| **Insomnia symptoms** |  |  |  |  |  | <0.001 |
| Never/Rarely | 96,258 (25%) | 6,749 (29%) | 4,521 (28%) | 82,608 (24%) | 2,380 (24%) |  |
| Sometimes | 188,040 (48%) | 11,178 (48%) | 7,662 (48%) | 164,571 (48%) | 4,629 (46%) |  |
| Usually | 107,495 (27%) | 5,308 (23%) | 3,895 (25%) | 95,219 (28%) | 3,073 (30%) |  |
| **Physical activity** |  |  |  |  |  | <0.001 |
| Inadequate | 100,474 (26%) | 4,584 (20%) | 3,732 (23%) | 89,010 (26%) | 3,148 (31%) |  |
| Adequate | 291,319 (74%) | 18,651 (80%) | 12,346 (77%) | 253,388 (74%) | 6,934 (69%) |  |
| **Dietary score** |  |  |  |  |  | <0.001 |
| 0 | 389 (0%) | 22 (0%) | 17 (0%) | 350 (0%) | 0 (0%) |  |
| 1 | 341 (0%) | 8 (0%) | 11 (0%) | 309 (0%) | 13 (0%) |  |
| 2 | 5,816 (1%) | 215 (1%) | 177 (1%) | 5,196 (2%) | 228 (2%) |  |
| 3 | 385,247 (98%) | 22,990 (99%) | 15,873 (99%) | 336,543 (98%) | 9,841 (98%) |  |
| **Body mass index, kg/m²** | 27.3 (4.7) | 22.2 (1.7) | 26.7 (3.2) | 27.6 (4.7) | 30.5 (5.1) | <0.001 |
| **Waist circumference, cm** | 89.8 (13.3) | 75.0 (7.3) | 85.5 (10.0) | 90.6 (12.9) | 102.2 (12.7) | <0.001 |
| **Systolic blood pressure, mmHg** | 137.8 (18.6) | 115.5 (8.3) | 117.5 (7.6) | 139.6 (17.4) | 161.2 (19.8) | <0.001 |
| **Diastolic blood pressure, mmHg** | 82.5 (10.1) | 70.7 (5.6) | 72.3 (5.1) | 83.6 (9.6) | 87.8 (11.3) | <0.001 |
| **HDL cholesterol, mg/dL** | 56.6 (14.7) | 65.0 (11.9) | 61.1 (11.2) | 56.1 (14.8) | 45.0 (10.6) | <0.001 |
| **Triglycerides, mg/dL** | 153.7 (90.4) | 80.9 (23.4) | 88.1 (23.8) | 160.0 (90.5) | 210.8 (115.5) | <0.001 |
| **Total cholesterol, mg/dL** | 223.1 (43.0) | 198.4 (24.5) | 201.8 (23.9) | 226.5 (43.5) | 198.9 (47.6) | <0.001 |
| **Fasting blood glucose, mg/dL** | 91.7 (21.0) | 83.6 (8.4) | 88.3 (11.0) | 91.7 (20.3) | 115.0 (45.7) | <0.001 |
| **HbA1c, %** | 5.4 (0.6) | 5.2 (0.3) | 5.3 (0.3) | 5.4 (0.6) | 6.3 (1.1) | <0.001 |
| **Serum creatinine, mg/dL** | 0.8 (0.2) | 0.8 (0.1) | 0.8 (0.1) | 0.8 (0.2) | 1.0 (0.6) | <0.001 |
| **C-reactive protein, mg/L** | 2.6 (4.3) | 1.2 (3.1) | 2.1 (3.9) | 2.6 (4.3) | 4.1 (5.7) | <0.001 |
| **eGFR, mL/min/1.73m²** | 95.6 (14.1) | 104.1 (11.4) | 100.7 (12.3) | 95.2 (13.7) | 80.5 (20.1) | <0.001 |
| **SPISE index** | 6.0 (1.8) | 8.7 (1.3) | 6.7 (1.3) | 5.9 (1.7) | 4.6 (1.3) | <0.001 |
| **Hypertension** | 300,010 (77%) | 0 (0%) | 0 (0%) | 289,962 (85%) | 10,048 (100%) | <0.001 |
| **Diabetes** | 37,794 (10%) | 0 (0%) | 0 (0%) | 29,585 (9%) | 8,209 (81%) | <0.001 |
| **Dyslipidemia** | 279,688 (71%) | 0 (0%) | 0 (0%) | 270,406 (79%) | 9,282 (92%) | <0.001 |
| **Metabolic syndrome** | 118,056 (30%) | 0 (0%) | 0 (0%) | 109,754 (32%) | 8,302 (82%) | <0.001 |
| **Antihypertensive medication** | 38,279 (10%) | 0 (0%) | 0 (0%) | 34,111 (10%) | 4,168 (41%) | <0.001 |
| **Statin use** | 6,892 (2%) | 0 (0%) | 0 (0%) | 6,737 (2%) | 155 (2%) | <0.001 |
| **Antidiabetic medication** | 1,915 (0%) | 0 (0%) | 0 (0%) | 1,693 (0%) | 222 (2%) | <0.001 |
| **Incident cardiovascular disease** | 67,397 (17%) | 1,202 (5%) | 951 (6%) | 60,399 (18%) | 4,845 (48%) | <0.001 |
| **Incident coronary heart disease** | 32,174 (8%) | 288 (1%) | 254 (2%) | 28,949 (8%) | 2,683 (27%) | <0.001 |
| **Follow-up years (CVD)** | 7.6 (4.0) | 8.5 (3.8) | 8.8 (3.7) | 7.7 (4.0) | 7.2 (4.1) | <0.001 |
| **Follow-up years (CHD)** | 7.7 (4.0) | 8.0 (4.0) | 8.2 (4.0) | 7.7 (4.0) | 7.1 (4.0) | <0.001 |
| ^1^Continuous variables are presented as mean ± standard deviation. Categorical variables are presented as n (%). For binary variables, values represent the number (percentage) of 'Yes' cases. | | | | | | |
| ^2^One-way analysis of means (not assuming equal variances); Pearson's Chi-squared test | | | | | | |

**Table S9. The performance of the SPISE index in Model 4 with CVD and CHD as the outcome**

|  | HR,95%CI | P value |
| --- | --- | --- |
| **CVD** |  |  |
| Per 1SD | 0.956 (0.947-0.965) | <0.001 |
| Per 1unit | 0.975 (0.970-0.980) | <0.001 |
| T1 (Low) | 1.00 (Reference) | NA |
| T2 (Middle) | 0.92 (0.90-0.93) | <0.001 |
| T3 (High) | 0.92 (0.90-0.94) | <0.001 |
| **CHD** |  |  |
| Per 1SD | 0.917 (0.904-0.931) | <0.001 |
| Per 1unit | 0.953 (0.945-0.961) | <0.001 |
| T1 (Low) | 1.00 (Reference) | NA |
| T2 (Middle) | 0.91 (0.89-0.94) | <0.001 |
| T3 (High) | 0.85 (0.82-0.88) | <0.001 |

Model4: Model3 + (insomnia status, comorbidities (diabetes, hypertension, hyperlipidemia), and CKM )

**Table S10. The performance of the SPISE index with CVD and CHD as the outcome after excluding events occurring within the first three years of follow-up**

|  | **Model 1** | | **Model 2** | | **Model 3** | |
| --- | --- | --- | --- | --- | --- | --- |
|  | HR,95%CI | P value | HR,95%CI | P value | HR,95%CI | P value |
| **CVD** |  |  |  |  |  |  |
| Per 1SD | 0.801 (0.794-0.809) | <0.001 | 0.818 (0.810-0.825) | <0.001 | 0.951 (0.941-0.961) | <0.001 |
| Per 1 unit | 0.884 (0.879-0.888) | <0.001 | 0.894 (0.889-0.898) | <0.001 | 0.972 (0.967-0.978) | <0.001 |
| T1 (Low) | 1.00 (Reference) | NA | 1.00 (Reference) | NA | 1.00 (Reference) | NA |
| T2 (Middle) | 0.75 (0.74-0.77) | <0.001 | 0.77 (0.76-0.79) | <0.001 | 0.79 (0.77-0.80) | <0.001 |
| T3 (High) | 0.64 (0.63-0.65) | <0.001 | 0.67 (0.65-0.68) | <0.001 | 0.69 (0.67-0.70) | <0.001 |
| **CHD** |  |  |  |  |  |  |
| Per 1SD | 0.714 (0.704-0.724) | <0.001 | 0.730 (0.720-0.741) | <0.001 | 0.918 (0.904-0.933) | <0.001 |
| Per 1 unit | 0.828 (0.822-0.835) | <0.001 | 0.839 (0.832-0.846) | <0.001 | 0.954 (0.946-0.962) | <0.001 |
| T1 (Low) | 1.00 (Reference) | NA | 1.00 (Reference) | NA | 1.00 (Reference) | NA |
| T2 (Middle) | 0.70 (0.68-0.72) | <0.001 | 0.73 (0.71-0.75) | <0.001 | 0.74 (0.72-0.76) | <0.001 |
| T3 (High) | 0.51 (0.49-0.53) | <0.001 | 0.53 (0.52-0.55) | <0.001 | 0.55 (0.53-0.57) | <0.001 |

**Table S11. The performance of the SPISE tertiles with CVD as the outcome after excluding participants with incomplete Data**

| Analysis type | HR,95%Cl | P value |
| --- | --- | --- |
| Complete-case(T2 vs T1) | 0.78  (0.77-0.80) | <0.001 |
| Complete-case(T3 vs T1) | 0.69  (0.68-0.70) | <0.001 |
| Multiple Imputation(T2 vs T1) | 0.79  (0.77-0.80) | <0.001 |
| Multiple Imputation(T3 vs T1) | 0.69  (0.68-0.70) | <0.001 |

**Table S12. The performance of the SPISE tertiles with CHD as the outcome after excluding participants with incomplete Data**

| Analysis type | HR,95%Cl | P value |
| --- | --- | --- |
| Complete-case(T2 vs T1) | 0.73  (0.71-0.74) | <0.001 |
| Complete-case(T3 vs T1) | 0.54  (0.52-0.56) | <0.001 |
| Multiple Imputation(T2 vs T1) | 0.73  (0.71-0.75) | <0.001 |
| Multiple Imputation(T3 vs T1) | 0.54  (0.52-0.56) | <0.001 |

**Table S13. The performance of the Continuous SPISE with CVD as the outcome after excluding participants with incomplete Data**

| Analysis type | HR,95%Cl | P value |
| --- | --- | --- |
| Complete-case(per 1 SD) | 0.833  (0.826-0.841) | <0.001 |
| Complete-case(per 1 unit) | 0.903  (0.899-0.908) | <0.001 |
| Multiple Imputation(per 1 SD) | 0.833  (0.826-0.840) | <0.001 |
| Multiple Imputation(per 1 unit) | 0.903  (0.899-0.907) | <0.001 |

**Table S14. The performance of the Continuous SPISE with CHD as the outcome after excluding participants with incomplete Data**

| Analysis type | HR,95%Cl | P value |
| --- | --- | --- |
| Complete-case(per 1 SD) | 0.735  (0.725-0.745) | <0.001 |
| Complete-case(per 1 unit) | 0.842  (0.836-0.849) | <0.001 |
| Multiple Imputation(per 1 SD) | 0.736  (0.726-0.746) | <0.001 |
| Multiple Imputation(per 1 unit) | 0.843  (0.836-0.849) | <0.001 |

**Table S15. Simplified PREVENT model for 10 year CVD**^10^

| **10 year Total CVD Risk** | |
| --- | --- |
| **Female** | **log-Odds** = -3.307728 + 0.7939329 × (age – 55) /10 + 0.0305239 × (Tc – HDL – 3.5) –0.1606857 × (HDL – 1.3) /0.3 – 0.2394003 × (min(SBP, 110) – 110) /20 + 0.360078 × (max(SBP, 110) – 130) /20 + 0.8667604 × (if diabetes) + 0.5360739 × (if current smoker) + 0.6045917 × (min(eGFR, 60) – 60) / -15 + 0.0433769 × (max(eGFR, 60) – 90) / -15 + 0.3151672 × (if using anti-hypertensive medication) – 0.1477655 × (if using statin) –0.0663612 × (if using anti-hypertensive medication) × (max(SBP, 110) – 130) /20 + 0.1197879 × (if using statin) × (Tc – HDL – 3.5) – 0.0819715 × (age – 55) /10 × (Tc – HDL – 3.5) + 0.0306769 × (age – 55) /10 × (HDL – 1.3) /0.3 – 0.0946348 × (age – 55) /10 × (max(SBP, 110) – 130) /20 – 0.27057 × (age – 55) /10 × (if diabetes) – 0.078715 × (age –55) /10 × (if current smoker) – 0.1637806 × (age – 55) /10 × (min(eGFR, 60) – 60) / -1  **Risk = exp(log-Odds) / (1 + exp(log-Odds))** |
| **Male** | **log-Odds** = -3.031168 + 0.7688528 × (age – 55) /10 + 0.0736174 × (Tc – HDL – 3.5) –0.0954431 × (HDL – 1.3) /0.3 – 0.4347345 × (min(SBP, 110) – 110) /20 + 0.3362658 × (max(SBP, 110) – 130) /20 + 0.7692857 × (if diabetes) + 0.4386871 × (if current smoker) + 0.5378979 × (min(eGFR, 60) – 60) / -15 + 0.0164827 × (max(eGFR, 60) – 90) / -15 + 0.288879 × (if using anti-hypertensive medication) – 0.1337349 × (if using statin) –0.0475924 × (if using anti-hypertensive medication) × (max(SBP, 110) – 130) /20 + 0.150273 × (if using statin) × (Tc – HDL – 3.5) – 0.0517874 × (age – 55) /10 × (Tc – HDL –3.5) + 0.0191169 × (age – 55) /10 × (HDL – 1.3) /0.3 – 0.1049477 × (age – 55) /10 × (max(SBP, 110) – 130) /20 – 0.2251948 × (age – 55) /10 × (if diabetes) – 0.0895067 × (age – 55) /10 × (if current smoker) – 0.1543702 × (age – 55) /10 × (min(eGFR, 60) – 60) / -15  **Risk = exp(log-Odds) / (1 + exp(log-Odds))** |

The PREVENT equations were applied to adults aged 30-79 years, with participants ≥80 years conservatively assigned an age of 79 years for risk estimation. Variables outside PREVENT-defined ranges (total cholesterol: 130-320 mg/dL; HDL: 20-100 mg/dL; systolic blood pressure: 90-200 mmHg; eGFR: 14-140 mL/min/1.73 m²) were truncated to boundary values

**Table S16. CKD-EPI equations for estimating GFR on the natural scale expressed for specified sex, serum creatinine or serum cystatin C**^11^

| Coefficients | Sex | Serum Creatinine (mg/dL) | Serum Cystatin C (mg/L) | Equation |
| --- | --- | --- | --- | --- |
| CKD-EPI creatinine-cystatin C equation | | | | |
| AS, new | Female | ≤0.7 | ≤0.8  >0.8 | GFR= 135×(Scr/0.7)^-0.219^×(Scys/0.8)^-0.323^×0.9961^Age^×0.963  GFR= 135×(Scr/0.7)^-0.219^×(Scys/0.8)^-0.778^×0.9961^Age^×0.963 |
|  |  | >0.7 | ≤0.8  >0.8 | GFR= 135×(Scr/0.7)^-0.544^×(Scys/0.8)^-0.323^×0.9961^Age^×0.963  GFR= 135×(Scr/0.7)^-0.544^×(Scys/0.8)^-0.778^×0.9961^Age^×0.963 |
|  | Male | ≤0.9 | ≤0.8  >0.8 | GFR= 135×(Scr/0.9)^-0.144^×(Scys/0.8)^-0.323^×0.9961^Age^  GFR= 135×(Scr/0.9)^-0.144^×(Scys/0.8)^-0.778^×0.9961^Age^ |
|  |  | >0.9 | ≤0.8  >0.8 | GFR= 135×(Scr/0.9)^-0.544^×(Scys/0.8)^-0.323^×0.9961^Age^  GFR= 135×(Scr/0.9)^-0.544^×(Scys/0.8)^-0.778^×0.9961^Age^ |

The CKD-EPI Creatinine-Cystatin C Age, Sex Equation (2021) can be expressed as a single equation 135×min(Scr/k,1)^α^×max(Scr/k,1)^-0.544^×min(Scys/0.8,1)^-0.323^×max(Scys/0.8,1)^-0.778^×0.9961^Age^×0.963 [if female] where Scr is serum creatinine Scys is serum cystatin C, k is 0.7 for females and 0.9 males, α is -0.219 for females and -0.144 for males, min indicates the minimum of Scr/k or 1, max indicates the maximum of Scr/k or 1.

**Figure S1. Analysis association of SPISE index and CHD incidencec in CKM syndrome stage 0–3**

Hazard ratio (HR) and 95% confidence intervals (CI) were estimated using multivariable Cox proportional hazards regression models. The model was adjusted for age, sex, race, education level, Townsend poverty index, alcohol consumption, smoking status, dietaryscore,lipid-lowering, antihypertensive, and antidiabetic drugs. SPISE, the Single-Point Insulin Sensitivity Estimator index. CKM, Cardiovascular-Kidney-Metabolic.

**Figure S2. Analysis association of SPISE tertiles and CVD incidencec in CKM syndrome stage 0–3**

**Figure S3. Analysis association of SPISE tertiles and CHD incidencec in CKM syndrome stage 0–3**

**Figure S4. Analysis association of SPISE quartiles and CVD incidencec in CKM syndrome stage 0–3**

**Figure S5. Analysis association of SPISE quartiles and CHD incidencec in CKM syndrome stage 0–3**
